# The Aryl Hydrocarbon Receptor and Its Crosstalk: A Chemopreventive Target of Naturally Occurring and Modified Phytochemicals

**DOI:** 10.3390/molecules29184283

**Published:** 2024-09-10

**Authors:** Hanna Szaefer, Barbara Licznerska, Wanda Baer-Dubowska

**Affiliations:** Department of Pharmaceutical Biochemistry, Poznan University of Medical Sciences, 3 Rokietnicka Street, 60-806 Poznań, Poland; barlicz@ump.edu.pl (B.L.); baerw@ump.edu.pl (W.B.-D.)

**Keywords:** AhR, ER, Nrf2, crosstalk, chemoprevention, *Brassica*, resveratrol, resveratrol analogs

## Abstract

The aryl hydrocarbon receptor (AhR) is an environmentally sensitive transcription factor (TF) historically associated with carcinogenesis initiation via the activation of numerous carcinogens. Nowadays, the AhR has been attributed to multiple endogenous functions to maintain cellular homeostasis. Moreover, crosstalk, often reciprocal, has been found between the AhR and several other TFs, particularly estrogen receptors (ERs) and nuclear factor erythroid 2-related factor-2 (Nrf2). Adequate modulation of these signaling pathways seems to be an attractive strategy for cancer chemoprevention. Several naturally occurring and synthetically modified AhR or ER ligands and Nrf2 modulators have been described. Sulfur-containing derivatives of glucosinolates, such as indole-3-carbinol (I3C), and stilbene derivatives are particularly interesting in this context. I3C and its condensation product, 3,3′-diindolylmethane (DIM), are classic examples of blocking agents that increase drug-metabolizing enzyme activity through activation of the AhR. Still, they also affect multiple essential signaling pathways in preventing hormone-dependent cancer. Resveratrol is a competitive antagonist of several classic AhR ligands. Its analogs, with ortho-methoxy substituents, exert stronger antiproliferative and proapoptotic activity. In addition, they modulate AhR activity and estrogen metabolism. Their activity seems related to a number of methoxy groups introduced into the stilbene structure. This review summarizes the data on the chemopreventive potential of these classes of phytochemicals, in the context of AhR and its crosstalk modulation.

## 1. Introduction

The cellular response to xenobiotic substances is mediated by the aryl hydrocarbon receptor (AhR) and its binding partner, ARNT [1]. Environmental contaminants like dioxins and polycyclic aromatic hydrocarbons (PAH) are the traditional ligands of AhR. Nevertheless, it is now known that a wide variety of endogenous and environmental ligands with different structural profiles can activate the AhR [2]. Earlier research has focused primarily on AhR-dependent xenobiotic metabolism. More recent studies have shown that the AhR is also involved in a wide range of other critical processes, such as cell differentiation, malignant transformation, immune response regulation, inflammation, and cell metabolism [3]. Although numerous studies have demonstrated the detrimental effects of ligands like PAH and dioxins that activate the AhR, AhR activation can also result in tumor suppression and immunomodulation [4]. Furthermore, there is ample evidence of the AhR’s interaction with transcription factors and other signaling pathways. The retinoblastoma protein (Rb), p53, nuclear factor NF-κB, nuclear factor erythroid 2-related factor-2 (Nrf2), estrogen receptors (ERs), and many others, are involved in these interactions [5]. The way the AhR and ERs interact has been particularly well-explained. Most evidence points to inhibitory AhR–ERα crosstalk, in which the activation of the AhR blocks ERα signaling, which in turn stops breast and endometrial cancer cells from proliferating and growing into mammary tumors [6]. The AhR and Nrf2 have well-tuned crosstalk that either mutually increases or lowers their activation statuses [7].

The AhR and its crosstalk may be involved in all stages of carcinogenesis through its impact on the regulation of cellular proliferation, the epithelial–mesenchymal transition (EMT), angiogenesis, stemness, and immune checkpoints. Therefore, the AhR has not only been associated with mammary and endometrial cancers mentioned above, but also several others, including T-cell leukemia, B-cell lymphoma, hepatocellular carcinoma, glioblastoma, and lung cancer [8]. Moreover, the association of AhR activity with increased tumor aggression and worse oncologic outcomes has been reported [9].

Numerous phytochemicals found in nature and their synthetic equivalents can alter AhR signaling, making them promising candidates for use as chemopreventive or therapeutic agents against cancer. Sulfur-containing breakdown products of glucosinolates (GLS) and stilbene derivatives are particularly noteworthy in this context.

Therefore, the aim of this review is to present the activities of these two classes of phytochemicals as modulators of AhR crosstalk and provide arguments on its importance as a chemopreventive and/or therapeutic target.

## 2. The Aryl Hydrocarbon Receptor: Mechanism of Transactivation, Physiological Functions and Their Role in Carcinogenesis

Several comprehensive reviews on the complex biology of the AhR have been published [2,10,11].

This section presents only an overview of the AhR functions related to cancer. The AhR is a member of the basic helix–loop–helix period/ARNT/single-minded protein family (bHLH_PAS family). The protein kinase SRC proto-oncogene, non-receptor tyrosine kinase (SRC), co-chaperone prostaglandin E synthase 3, the AhR interaction protein, and two 90 kDa heat-shock proteins, form a complex with the AhR in its inactive state, as it appears in the cytoplasm [12]. Ligand binding exposes its nuclear localization sequence and allows translocation into the nucleus, where the AhR forms a heterodimer with the AhR nuclear translocator (ARNT) and binds to the DNA sequence of xenobiotic response elements (XREs) leading to AhR target gene expression. Following transcriptional activation, the AhR is exported from the nucleus into the cytoplasm and degraded by ubiquitin-dependent proteasome pathway. Canonical AhR signaling is thought to occur when the AhR–ARNT complex binds to XREs [2]. Initially, it was believed that planar molecules with hydrophobic structures were the only ones that could activate the AhR. Consequently, the AhR’s capacity to trigger the transcription of multiple genes encoding enzymes involved in the metabolism and bioactivation of carcinogens, specifically PAH, polychlorinated biphenyls, and dioxins, such as 2,3,7,8-tetrachloro-dibenzo-p-dioxin (TCDD), has attracted a great deal of research attention [13].

The AhR activation results in the induction of cytochrome P450 enzymes, such as CYP1A1, CYP1A2, and CYP1B1, which convert inert parent chemicals into electrophilic reactive intermediates and form DNA adducts. These adducts have the potential to initiate the carcinogenesis process [14]. AhR target genes include those that encode phase 2 enzymes, such as glutathione-S-transferase (GST, i.e., GSTA1), epoxide hydrolase 1 [12], and UDP-glucuronosyltransferases (UDGTs) [15], in addition to CYPs.

Through the production of reactive oxygen species (ROS) and the upregulation of proinflammatory cytokines, AhR activation also causes oxidative stress [16].

Therefore, AhR modulation has been suggested as a cancer chemoprevention strategy, since the pathways controlled by this transcription factor are critical in the beginning and during the development of cancer [17].

Nevertheless, DNA microarray investigations have shown that the AhR either directly or indirectly controls the expression of a wide range of genes involved in several metabolic pathways, such as lipid and cholesterol synthesis, energy metabolism, xenobiotic metabolism, and numerous transporters [18]. Therefore, AhR expression/activation may impact the migration, proliferation, and survival of cancer cells [19,20]. Furthermore, different AhR ligands may activate this receptor in various ways [13], and it has been proposed that distinct AhR binding pockets may have distinct consequences and may influence canonical AhR activation [2]. As a result, AhR ligands have been shown to have extremely tumor-specific actions that can either promote or inhibit carcinogenesis. As a result, agonists and antagonists of selective AhR modulators are regarded as a significant class of anticancer drugs [21].

In addition to the genomic regulation of target genes’ expression, several non-genomic effects of the AhR have been identified [2]. In this regard, the AhR in the cytoplasm interacts with a non-receptor tyrosine kinase, SRC [22]. Different ligands that activate the AhR cause SRC to become phosphorylated, which activates the downstream proteins, including the epidermal growth factor receptor (EGFR). Additionally, it has been observed that cytoplasmic AhR interacts with a signal transducer and activator of transcription (STAT1) [23], which may be crucial for the development of negative inflammatory responses [24].

The AhR activation, translocation, and DNA binding are regulated by covalent modification, particularly by phosphorylation, of the AhR itself and its interaction partners. Consequently, the AhR response to various stimuli, including carcinogens, may be influenced by the expression and activation of kinases implicated in this process in different cell types. Furthermore, the methylation of the AhR gene promoter, histone modification, and microRNA targeting are among the epigenetic factors/modifications that are crucial in controlling AhR expression and its target genes [2]. The epigenetic silencing of the AhR itself or its downstream targets after exposure to food or the environment may modify the redox balance, impact carcinogen removal, or lessen activation of the carcinogens. In addition, AhR/CYP1A1 pathway-mediated epigenetic mechanisms play a significant role in regulating gene expression, self-renewal, and the chemoresistance of cancer stem cells in a variety of human malignancies [25]. Thus, the epigenetic modification of the AhR itself or its involvement in the epigenetic mechanism may contribute to cancer development.

The high degree of complexity emerging from AhR regulation and crosstalk with several signaling pathways contribute to its dual role in cancer. Furthermore, various classes of AhR ligands, and even ligands within the same class, can differently modulate the AhR and, as a consequence, the tumorigenic outcomes, i.e., tumor-specific suppressive or promoter-like activities [26]. However, the AhR and its interactions with other signaling pathways, including Nrf2, and ERs make it a potentially significant target for cancer treatment and chemoprevention, especially concerning naturally occurring phytochemicals and their derivatives.

## 3. The AhR’s Interactions with Nuclear Factor Erythroid 2-Related Factor-2

As a member of the cap‘n’collar (CNC) subfamily, Nrf2 regulates the oxidative and xenobiotic stress response pathway, which is crucial for preventing DNA damage from reactive electrophilic or oxidative species and mutagenic events that could result in the development of cancer [27]. Nrf2 is an important element of cellular homeostasis by inducing the expression of more than 250 target genes that control oxidation/reduction processes, the metabolism of xenobiotics, DNA repair, and carbohydrate and lipid metabolism, in addition to ensuring proteostasis [28].

The cytoprotective activity of Nrf2 has been implicated in disease prevention, particularly in the blocking of the initiation stage of cancer development. On the other hand, several recent reports have indicated that Nrf2 is upregulated in different types of tumors and that its enhanced activity correlates with tumor progression, aggressiveness, resistance to therapy, and poor prognosis [29]. Therefore, both the inducers and the inhibitors affecting different elements of the Nrf2–ARE pathway may be considered as potential chemopreventive agents or as therapeutics in cancer and other diseases, respectively.

There are two other members of the Nrf subfamily, Nrf1 and Nrf3, in addition to Nrf2 [30]. The first one, Nrf1, is a transcription factor that regulates the expression of nuclear genes necessary for respiration, heme production, mitochondrial DNA transcription and replication, and several important metabolic genes controlling cellular growth [31]. The second one, Nrf3, is important for cancer function. Accordingly, a poor prognosis is linked to elevated Nrf3 mRNA levels, which are activated in various cancer types, including pancreatic adenocarcinoma and colorectal cancer [32]. The canonical and the non-canonical are the two fundamental Nrf2 activation pathways that have been identified. Canonical activation is based on the dissociation/release of Nrf2 from its inactive complex with the repressor protein Keap1 in the cytoplasm and the subsequent translocation of Nrf2 into the nucleus. Under normal conditions, this complex is recruited to the ubiquitin ligase complex, leading to Nrf2 degradation in the proteasome. In stressful circumstances, a conformational shift brought about by the modification of important cysteine residues in Keap1 inhibits Nrf2 ubiquitination, allowing its translocation into the nucleus, where it binds to an antioxidant response element (ARE) and activates the transcription of the target genes, including GST, UDGT, NAD(P)H:quinone oxidoreductase (NQO1), heme-oxygenase-1 (HO-1), and glutathione peroxidase (GPx) [33,34]. In regard to the non-canonical Nrf2 activation pathway, the release of Nrf2 from its complex with the Keap1 inhibitor results from the interaction of Nrf2 or Keap1 with other proteins, such as p62, DPP3, WTX, prothymosin α, PALB2, and p21. Through these interactions, Nrf2 is shielded from ubiquitination and the breakdown of 26S proteasomes, enabling it to translocate into the nucleus.

The most studied mechanism involving the non-canonical pathway is the activation of Nrf2 by the p62 protein (SQSTM1)*,* an autophagy receptor. The Nrf2 activation dependent on p62 increases the expression of not only NQO1 and GST, but also antiapoptotic proteins, such as Bcl-2 and Bcl-xL, decreasing ROS levels and protecting the cell against oxidative stress [35,36]. However, sustained Nrf2 activation by an impairment in autophagy and an increase in p62 phosphorylation promotes antineoplastic drug chemoresistance, as well as cancer cell proliferation, whereas a mutation in the KIR domain in p62, which prevents a Keap1–p62 interaction, is associated with an ROS increase [37,38,39,40,41,42].

Similarly, as in the case of the AhR, epigenetic mechanisms have been implicated in the complex regulation of the Nrf2–Keap1 pathway. Furthermore, the deregulation of these mechanisms is frequently seen in cancer [43]. In this context, several microRNAs have been linked to the suppression of Nrf2 expression. Along with the mutation of Nrf2 or Keap1, the downregulation of miR-507, miR-634, miR-450A, and miR-129-5p increased the stabilization of Nrf2, which was linked to poorer survival and distant metastases in esophageal carcinomas [44]. By lowering the amount of Nrf2 mRNA, the overexpression of miR-28 in breast cancer cells decreased the Nrf2 function in a Keap1-independent way [45].

**Figure 1:**
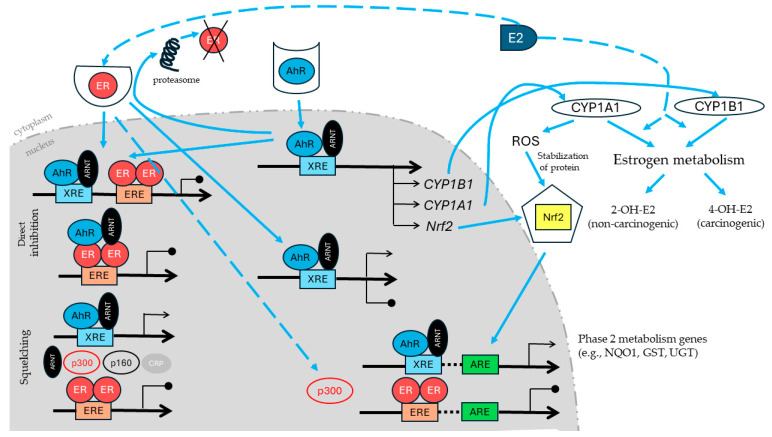
The outline shows the selected possible AhR–ER–Nrf2 crosstalk. The presented mechanisms, including different gene expression regulation via XRE, ERE, and ARE, increased ROS production, and estrogen metabolism are discussed in detail in the text. AhR—aryl hydrocarbon receptor, ARE—aryl hydrocarbon response element, ARNT—AhR nuclear translocator, CRP—cyclic AMP receptor protein, E2—β-estradiol, ER—estrogen receptor, ERE—estrogen response element, GST—glutathione-S-transferase, NQO1—NAD(P)H:quinone oxidoreductase, Nrf2—nuclear factor erythroid 2-related factor-2, p300—transcriptional coactivator p300, p160—transcriptional coactivator p160, ROS—reactive oxygen species, UGT—UDP glucuronosyltransferases, XRE—xenobiotic response elements, 2-OH-E2—2-hydroxy-estradiol, 4-OH-E2—4-hydroxy-estradiol.

There is fine-tuned crosstalk between AhR and Nrf2, which mutually enhances or weakens their activation states (Figure 1) [7,46]. Moreover, there is an overlap between Nrf2 and AhR target genes, like NQO1, GSTA2, and UDPT1A6. The promoters of these genes contain functional XREs and AREs. Therefore, the induction of these genes requires activation of the AhR and Nrf2 [47]. Two mechanisms of AhR–Nrf2 interactions have been proposed [48,49]: (1) the direct transcription activation of Nrf2 or AhR genes through AhR binding to XRE in the Nrf2 promoter or Nrf2 to the CNC–sMAF binding element in the AhR promoter; (2) an indirect interaction through the generation of ROS by the induction of CYP1A1. So far, it is unclear which of these two mechanisms of AhR–Nrf2 predominates. The existing data support both pathways [48]. Many phytochemicals affect these pathways, acting as Nrf2 agonists with AhR agonistic activity, Nrf2 agonists with AhR antagonistic activity, or Nrf2 agonists with CYP1A1 inhibitor activity. The latter mechanism refers, among others, to naturally occurring stilbenes, resveratrol, and pterostilbene [7].

Therefore, it is conceivable that a simultaneous modulation of AhR–Nrf2 signaling pathways exerts chemopreventive effects.

## 4. Estrogen Receptor α and β and Reciprocal AhR–ER Crosstalk

Estrogens, particularly 17β-estradiol (E2), regulate several physiological processes and are implicated in the pathogenesis of hormone-dependent cancers, such as breast, endometrium, and ovarian cancers [50]. The biological activity of estrogens is mediated by their binding to nuclear estrogen receptors, ERα and ERβ [51,52]. These two ER subtypes are transcribed from different genes located on separate chromosomes and display discrete expression patterns and distinct ligand specificities [53,54]. ERs, like other nuclear receptors, have a modular structure consisting of separate functional domains. ERs can activate gene expression by binding to specific recognition sites in the regulatory regions of target genes, either directly or indirectly, through protein–protein interactions. ERs have two transactivation domains, AF-1 and AF-2. The amino terminal AF-1 differs substantially in human ER subtypes [54]. In contrast, DNA-binding domains are highly conserved at the amino acid level and the receptors bind to the same DNA sites, i.e., estrogen response elements (EREs), either as homodimers or heterodimers. The ligand-binding domain contains the AF-2 domain. The synergy between AF-1 and AF-2 leads to full transcriptional activity of the ERs [55]. Besides these classical ERs, the membrane-localized G protein-coupled estrogen receptor 1 (GPER1) is also involved in estrogen action. GPER1 plays an important role in the rapid signaling events following cell and tissue stimulation with estrogen and other stimuli [56]. ERα and ERβ have similar affinities for E2 and display overlapping and distinct expression patterns in different tissues [57]. ERα mediates most estrogen signaling in classic estrogen targets, such as the mammary gland, where ERβ plays a minor role. Therefore, ERα is considered the most important target of chemopreventive and therapeutic agents in breast cancer [58].

Crosstalk between AhR and ER signaling, mainly ERα, is well documented (Figure 1).

The interaction between these signaling pathways in breast cancer relates to how estrogens may contribute to breast cancer development. One is the induction of proliferation via ER, while the other is the generation of reactive metabolites of estrogens, such as quinones, as well as the formation of ROS produced in reactions catalyzed by different CYPs, particularly the CYP1 family, whose expression is controlled by the AhR [59]. The AhR not only activates the expression of genes metabolizing estrogens, but also promotes the degradation of ER [60]. Therefore, it can be assumed that the modulation of the AhR and, subsequently, the expression of genes controlled by this receptor, may depend on the estrogen receptor status of breast cells.

However, despite many studies, the mechanisms of reciprocal AhR–ER interactions have not been entirely explained. While, in most cases, the inhibitory effects of the AhR on ERα were observed, the AhR-mediated estrogenic effects were also described [61,62]. The impact of ERα on AhR signaling differed from inhibition to activation or no effect [63,64,65].

Numerous mechanisms of crosstalk between AhR and ER have been proposed: (1) well-established, increased estrogen metabolism via extended CYP1A1 and 1B1 pathways; (2) the direct inhibition by the activated AhR/ARNT heterodimer through binding to the inhibitory XRE present in ER target genes; (3) the squelching of shared coactivators, including ARNT; (4) the increased proteasomal degradation of ER,; (5) the synthesis of an unknown inhibitory protein [62].

Swendenberg and Pongratz [66] investigated ARNT’s role as an ER modulator. They suggested that ARNT mediated or stabilized the interactions between ER and cofactor p300, which had a strong histone methyltransferase activity and mediated the synergy between the two trans-activations. Interestingly, ARNT had a much more substantial impact on Erβ, than Erα, activity. This could be due to the ERβ AF-1 domain, which is weaker than ERα [66]. A complex AhR/ERα crosstalk at the transcriptional level was shown in the human hepatoma cell line HepG2 and indicated that TCDD acted as anti-estrogen via downregulation of E2-mediated ERα signaling. Different responses in HepG2 cells compared to cells derived from hormone-regulated tissues may suggest that the involved molecular mechanisms of the ER and AhR signaling differ in a cell- or tissue-dependent manner, such as the receptor levels or available co-regulatory proteins that may interact with the receptors [67].

In summary, most of the data support inhibitory AhR–ERα crosstalk, where the activation of the AhR inhibits ERα signaling, leading to the inhibition of breast and endometrial cancer cell proliferation, survival, migration/invasion, and metastasis. However, AhR agonists or antagonists may exert opposite effects due to the cell context and ligand structure [68]. The complexity of crosstalk between AhR and ERα requires consideration in searching for new chemopreventive or therapeutic agents, particularly for treating hormone-dependent cancers.

## 5. Targeting AhR Crosstalk with ER and Nrf2 through Naturally Occurring and Modified Phytochemicals in Chemoprevention

**Figure 2:**
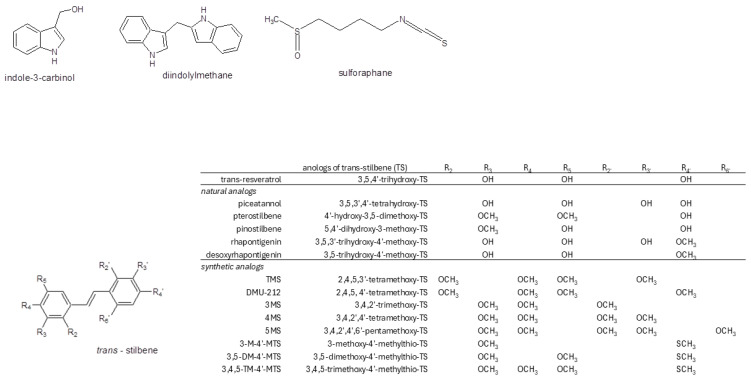
The structures of the described *Brassica* phytochemicals, resveratrol, and its analogs.

*Brassica*-derived phytochemicals and naturally occurring stilbenes have been the subject of intensive studies, showing their chemopreventive potential at the different stages of carcinogenesis. While the increasing weight of clinical evidence suggests their beneficial effects on human health, high-quality clinical trials including new ways of delivery are still required to confirm their potential application in clinics [69]. One of the most interesting aspects of their possible application in this context is the support of conventional anticancer therapy. In this regard, the combinational use of ITCs with chemotherapeutic drugs, such as cisplatin and taxanes, have enhanced the effect of the latter and pointed out a potential way to improve therapy outcomes [70]. The chemosensitizing effects of resveratrol on several anticancer drugs have also been recently reviewed [71]. Taking into consideration the current knowledge on the impact of the AhR and its crosstalk on carcinogenesis, certainly its modulation by the phytochemicals described in this section (Figure 2) plays an important role in their ultimate effects.

### 5.1. Brassica-Derived Phytochemicals Targeting AhR–ER and AhR–Nrf2 Crosstalk

*Brassica* vegetables, members of the *Brassicaceae* family, also known as Cruciferae, are rich sources of sulfur-containing GSLs, such as glucobrassicin and glucoraphanin. These compounds contain a common glycone moiety and a variable side chain derived from amino acids. When plant tissue is disrupted, an endogenous plant β-thioglucoside glucohydrolase (myrosinase) catalyzes the GLS degradation, initially to unstable thiohydoximate-O-sulfonate, spontaneously rearranging to isothiocyanates (ITCs) or indoles [72]. The pioneering experiments by Wattenberg and Loub in 1978, showing the tumor-inhibitory effects of various indoles [73], aroused great interest in cruciferous vegetables and their active components, particularly I3C, the products of its condensation, and various ITCs, for chemoprevention purposes. Among the mechanisms of chemopreventive action of these compounds, the modulation of the metabolism of exogenous and endogenous carcinogens, resulting from their effects on AhR and Nrf2 activation and the subsequent induction of CYPs and phase 2 enzymes, was the major subject of investigation, along with their impact on ERα.

#### 5.1.1. Indoles

Among the *Brassica*-derived indoles, I3C and its condensation product, 3,3′-dimethylmethane (DIM), have been the subject of the most intensive studies. I3C affects multiple signaling pathways and targets molecules controlling cell division, apoptosis, or angiogenesis deregulated in cancer cells [74]. I3C is known as a strong AhR agonist [75] in contrast to DIM, which is considered a weak agonist of this receptor [76]. Consequently, as early studies showed, DIM was a markedly less efficacious inducer of CYP1, in general, and of its specific isoforms in rat models at concentrations relevant to human supplementation [77]. The analysis of metabolic profiles in these early studies indicated the increased metabolism of food-derived carcinogens through the induction of CYP1A1 and CYP1A2 [72]. However, in some systems, for e.g., human T47-D breast cancer cells, both I3C and DIM reduced CYP1A1 activity [78]. Recently, based on experiments in cervical cancer cells (HeLa) and cervical cancer samples, Arellano-Gutiérrez and co-workers have suggested that I3C, through the activation of the AhR, induces UBE2L3 transcription, which might result in the ubiquitination of HPV E7, an element of human papillomavirus and, ultimately, in decreasing cell proliferation [79].

Various phase 2 enzyme activities were enhanced by I3C, but to a lesser extent than AhR-dependent CYPs. However, the data obtained from the study using an Nrf2 knockout model indicate that I3C regulates intestinal GST expression through an Nrf2-dependent mechanism, whereas I3C’s effect on NAD(P)H:quinone oxidoreductase (NQO) involves another mechanism [80]. In our previous studies, the increased expression of phase 2 detoxifying enzymes (e.g., GSTs and NQO1) by I3C and DIM in breast cancer cell lines correlated with the upregulation of Nrf2 [81]. Thus, indoles may activate the Nrf2-dependent pathway, directly or via the upregulation of the AhR [82].

The agonistic effect of I3C toward the AhR is related to the downregulation of ERα and the expression of estrogen-responsive genes [83]. In MCF7 and T47D breast cancer cells expressing both ERα and ERβ, I3C strongly reduced the ERα transcript and protein levels without altering the ERβ protein [84]. Moreover, Marconett and co-workers have shown that I3C could be involved in ERα/GATA3 cross-regulatory feedback loop disruption via the induction of the ubiquitination and proteasome-mediated degradation of ERα, resulting in estrogen-dependent growth arrest [85].

DIM, a weak agonist of AhR, affects AhR–ER crosstalk differently, depending on the cell type and the concentration [76]. This compound may induce estrogen responses via a ligand-independent ER activation or modulate estrogenic responses through AhR interactions [86]. In particular, in breast and endometrial cancer cells, DIM in high doses (50 µM) has shown antiestrogenic activity, while in lower concentrations (10µM), the upregulation of ERα by this compound has been noticed [87,88]. In addition, DIM-related ERα protein degradation has been observed in breast cancer cells [86]. In a few other cell types, the formation of the DIM–AhR complex with ERα has provoked the activation of some estrogen-responsive gene promoters [89]. In our studies, I3C and DIM have simultaneously upregulated AhR and downregulated ERα expression in the ER-positive MCF7 cell line [81]. DIM can interfere with E2 metabolism, since the target genes for DIM-dependent AhR activation are CYP1A1 and CYP1B1, effective in terms of estrogen hydroxylation at the 2- and 4-position, respectively. The ultimate result of DIM-dependent E2 metabolism modification is a reduction in the 16α/2-hydroxy-E2 in the urine. A subsequent decrease in the E2 level [90,91] can contribute to a beneficial effect of this indole. Our earlier studies have shown the induction of CYP1A1 and CYP1B1 in different breast epithelial cell lines treated with both indoles [92]. The ratio of CYP1A1/CYP1B1 induction by indoles was 10-magnitude in favor of noncancerous 2-hydroxy metabolites mediated by CYP1A1 activity [92].

It should be noticed that AhR knockout mice did not show any I3C-related upregulation of estrogen 2-hydroxylation and several CYP1A inducers did not influence this process. Thus, it was concluded that 2-hydroxylation of estrogens depends on the AhR, but not on CYP1A1 activity [93]. Interestingly, we demonstrated the increased AhR expression in the ER-negative MDA-MB-231 cell line [81]. Since the upregulation of CYP1A1 may lead to increased ER expression, the induction of the AhR in ER-negative cells may potentially increase the ER level in these cells and, thus, sensitize them in regard to conventional hormone therapy [94,95].

On the other hand, the results of some early studies [96] showed that DIM inhibits the proliferation of both estrogen-dependent (MCF7) and triple-negative MDA-MB-231 breast cancer cells, pointing out the induction of p21 protein expression as an underlying mechanism.

Therefore, it is clear that DIM activity in breast cancer may be due, in part, to inhibitory AhR–ER crosstalk, but multiple growth inhibitory/cell death pathways impacting breast and other cancer cell lines induced by DIM are AhR-independent [97]. Similar conclusions have been drawn from the studies on ring-substituted DIMs, such as 1,1-*bis*(3′-indolyl)-1-(*p*-substituted phenyl)methanes). Recently, DIM-biaryl conjugates have been tested against MDA-MB-231, showing their antiapoptotic effect. However, their effect on AhR–ER crosstalk has not been evaluated [98].

Although most research on the cellular effects of GSL derivatives has primarily focused on the enzymes involved in carcinogen metabolism, increasing evidence has demonstrated the chemopreventive effects of dietary GSL derivatives on the epigenetic regulation of silenced genes, including which products regulate the expression of AhR, Nrf2, and ERs. In this regard, DIM has been shown to suppress the expression of HDAC2 and HDAC3 proteins in mouse prostate cancer TRAMP-C1 cells, with a concomitant increase in apoptosis, a decrease in cell proliferation, and enhanced Nrf2 and Nrf2 target gene NQO1 expression [99].

#### 5.1.2. Isothiocyanates

The most extensively studied ITCs are sulforaphane (SFN) and phenethyl-isothiocyanate (PEITC). Recently, moringa isothiocyanates (MIC) have attracted the attention of researchers. These compounds differ in structure and in terms of their physical properties [100], but all induce phase 2 enzymes, particularly GSTs, NQO, and UDPGT. The interaction of ITCs with Nrf2 results in the induction of these enzymes. It is claimed that SFN, PEITC, and MIC have a high affinity for Keap1. However, the interaction of ITC with this Nrf2 inhibitor seems to depend on many factors, such as the cell type, concentration, and exposure, etc., which still need to be well-characterized [101]. PEITC and SFN have been shown to activate Nrf2, both in vitro and in vivo. In this regard, our early study demonstrated that treatment with PETIC significantly increased GST (particularly µ and α isoforms) and NQO1 in rat liver and kidney, causing the translocation of Nrf2 from the cytoplasm into the nucleus, indicating Nrf2 activation [102]. Several other studies, including ours, confirmed this effect in different models [103]. It was suggested that Nrf2 activation by PEITC may result from the increased phosphorylation of extracellular signal-regulated kinases 1/2 (ERK1/2), responsible for Nrf2 phosphorylation and translocation into the nucleus [104]. The induction of Nrf2 signaling, along with the inhibition of NF-κB, was also found in the case of MIC. The NF-κB transcription factor is involved in inflammation. Therefore, MIC and other ITCs may exert anti-inflammatory effects [105].

Our study on ER-positive and ER-negative breast cancer cells, as well as nontumorigenic breast cells, showed the increased expression of GSTP after treatment with R-SFN (a naturally occurring L-isomer of SFN). Increased NQO1 transcript and protein levels were found in all breast cells, with the most significant increase in ER-positive MCF7 cells. Similarly, the enhancement of Nrf2 expression was noticed in all tested cell lines [106].

The effects of ITCs on the expression and activity of AhR-controlled CYPs are ambiguous. SFN reduced the CYP1A1 protein level equally in nontumorigenic MCF10A breast cells, ER-positive MCF7, and ER-negative MDA-MB-231 breast cancer cells, but the increased level of CYP1A2 and the decreased level of CYP1B1 expression were found only in MCF10A cells [107]. AhR gene expression was decreased merely in MCF7 cells [106].

Glucoraphanin, a main source of SFN, strongly induced CYP1A1, 1A2 in rat lungs, while the GST activity was only slightly induced [108]. The inhibitory effect of SFN on B[a]P-induced AhR mice, with enhanced Nrf2 activation, was described by Kalpana and co-workers [109], suggesting its anti-initiating activity in mouse lung carcinogenesis.

To sum up, interference by *Brassica* GSL degradation product indoles and ITCs with the AhR–Nrf2–ER axis certainly contributes to the anticarcinogenic effects observed in pre-clinical studies, particularly in hormone-dependent cancers. While some effects, like in the case of SFN-induced Nrf2 activation, might be beneficial in breast cancer prevention, others, like the induction of GSTP, may lead to adverse effects. The target tissue, concentration, and possible synergism of indoles and ITCs must be considered in the chemopreventive application of these compounds.

### 5.2. Resveratrol and Its Natural Synthetic Analogs Targeting AhR–ER and Nrf2 Crosstalk

#### 5.2.1. Resveratrol

A naturally occurring phytoalexin, resveratrol (3,5,4′-trihydroxy-trans-stilbene), is present in numerous plants, but it is most commonly found in red grape skin. Jang et al. originally reported in 1997 the inhibitory effect of resveratrol on all stages of carcinogenesis [110]. Subsequently, numerous studies, both in vitro and in vivo, have demonstrated its capacity to modulate many signaling pathways associated with cellular growth and division, apoptosis, migration, and invasion [111]. Resveratrol’s ability to suppress AhR-mediated CYP enzyme activation has been shown in several studies [112].

In MCF7 cells, resveratrol inhibited the recruitment of AhR and ARNT to CYP1A1/CYP1B1 and CYP1A1/1A2 promoters, which was increased by TCDD. AhR-regulated transcription was inhibited by resveratrol in both ER-dependent and ER-independent ways. However, resveratrol’s ability to suppress AhR activity is concentration-dependent. At nanomolar concentrations, resveratrol might block AhR-mediated CYP1A1 induction in an ERα-dependent manner without diminishing the level of the functional transcriptional factor complex, the AHRC at the CYP1A1 promoter. In contrast, AHRC on the CYP1A1 promoter was significantly reduced by 10 pM of resveratrol, leading to the nearly total suppression of CYP1A1 expression and metabolic activity [112,113]. On the other hand, MacPherson and Matthews [114] showed that the RNAi-mediated knockdown of ERα in T-47 D human epithelial breast cancer cells did not affect the inhibitory action of resveratrol on AhR activity. Furthermore, in ER-negative MDA-MB-231 and BT-549 breast cancer cells, resveratrol (10 µM) suppressed AhR-dependent transcription, suggesting that ERα was not involved in resveratrol’s AhR-inhibiting effects. Resveratrol stimulated ERα expression and recruitment to GREB1 during the same investigation. This gene, which is significant in hormone-responsive tissues and cancer, is an early responder gene in the ER regulation pathway [115].

Other authors have confirmed the relationship between the effect of resveratrol on AhR and ER, according to resveratrol’s concentration. In this context, it was demonstrated that resveratrol at high concentrations acted as an antiestrogen in in vitro and in vivo models derived from endometrial adenocarcinoma Ishikawa cells and in human endometrium, respectively. In Ishikawa cells, resveratrol itself seems to have a negligible estrogenic effect. However, resveratrol functions as an estrogen antagonist at high concentrations (100 µM) and an estrogen agonist at low concentrations (1 and 10 pM) when estrogen is present [116].

An increased activity of AhR compared to the effect of benzo[a]pyrene and indigo at low concentrations of resveratrol (up to 50 nM) was reported in mouse hepatocytes. Conversely, when benzo[a]pyrene or indigo were present, resveratrol functioned as an AhR inhibitor at higher concentrations (~200 nM) [117].

Corre et al. [118] showed that the AhR was constitutively activated in a subpopulation of melanoma cells, which promoted BRAFi-resistance genes and melanoma cell dedifferentiation. Resveratrol abrogated deleterious AhR, sustained activation in these cells, and reduced the number of BRAFi-resistant cells, thus delaying tumor growth.

As was mentioned in the introduction section, AhR may regulate EMT. Therefore, it is interesting to note that resveratrol is able to reverse EMT in some cancer cells and through this overcome doxorubicin resistance [119,120].

Several studies have described the modulation of Nrf2 transcription factors, and the induction of phase 2 enzymes controlled by this transcription factor through resveratrol. Additionally, it has been demonstrated by Larigot et al. [13] that resveratrol increases the expression of paraoxonase 1, but not CYP1A1, in human hepatocellular cells, perhaps facilitating the detoxification of oxidized lipids. The authors speculated that it might be because the AhR binds to different XREs. Resveratrol protected primary hepatocytes exposed to oxidative stress via increased Nrf2-mediated NQO1, catalase, superoxide dismutase, glutathione reductase, glutathione peroxidase, and glutathione-S-transferase expression [121]. Furthermore, resveratrol increased NQO1 expression and activity in the K562 leukemia cell line, which was associated with resveratrol-induced Nrf2/Keap1 complex disruption, Nrf2 nuclear translocation, and subsequent binding to ARE within the NQO1 promoter [122].

In cigarette smoke extract-treated bronchial epithelial cells, resveratrol-induced Nrf2 signaling led to the enhanced expression of NQO1, heme-oxygenase 1 (HO-1), and the glutamate cysteine ligase catalytic subunit [123]. Resveratrol was shown by Kode et al. [124] to restore glutathione levels in A549 lung alveolar epithelial cancer cells treated with cigarette smoke extract; this effect was mediated by Nrf2-induced glutamate cysteine ligase expression and activity through the inhibition of Nrf2-modified Nrf2 post-translation. Resveratrol’s induction of NQO1 expression in TCDD-treated MCF10F immortalized breast cells involved Nrf2, suppressing DNA adduct formation [125]. Following treatment with 4-hydroxy-E2 and E2-3,4-quinone, resveratrol also enhanced NQO1 [126].

According to our research, resveratrol enhanced Nrf2 activation, which led to an increase in the expression of the genes SOD, GSTP, NQO1 CAT, and GPx in human pancreatic cancer cells MIA-Pa-Ca-2 and hepatocellular carcinoma HepG2 cells [127,128].

K562 leukemia and HepG2 cell lines exhibited Nrf2 cytoplasmic accumulation and resveratrol-induced suppression of Nrf2-dependent transcription. This suggests that resveratrol may impact Nrf2 translocation and accumulation [129]. Therefore, the effect of resveratrol on the AhR–Nrf2–ER axis is not ambiguous.

Epigenetic processes can be altered by resveratrol [130,131]. Resveratrol’s involvement in epigenetic alterations potentially influences AhR–Nrf2–ER crosstalk and, in turn, the process of carcinogenesis. For example, in a study evaluating the effects of resveratrol on E2-induced carcinogenesis [132], resveratrol upregulated Nrf2 in the mammary tissues of rats and attenuated the repressive effects of E2 on Nrf2. E2 inhibited Nrf2 through DNA methylation, but resveratrol reversed this effect. Resveratrol treatment inhibited E2-mediated changes in Nrf2 promoter methylation and Nrf2 targeting miR-93 expression, suggesting that resveratrol regulated Nrf2’s epigenetic expression in E2-induced breast carcinogenesis. The recruitment and activation of the AhR at the BRCA-1 promoter inhibits the transcriptional and protein levels of BRCA-1 in MCF7 cells when stimulated by E2. The idea that resveratrol could prevent the epigenetic silencing of the BRCA-1 gene by preventing the AhR repression of BRCA-1 expression, which happened as a result of resveratrol pretreatment, is supported by the AhR-dependent activation [133].

#### 5.2.2. Resveratrol Naturally Occurring Analogs

In addition to resveratrol itself, its natural analogs, such as pterostilbene and piceatannol, the product of resveratrol metabolism, were the subject of studies evaluating their potential chemopreventive activity related to AhR modulation. Our preliminary study revealed that naturally occurring trans-resveratrol analogs were significantly more effective at inhibiting human recombinant CYP1A1 than the parent compound. These analogs were pinostilbene (3,4′-dihydroxy-5-methoxystilbene), desoxyrhapontigenin (3,5-dihydroxy-4′-methoxystilbene), and pterostilbene (3,5-dimethoxy-4′-hydroxystilbene). In contrast, pterostilbene, desoxyrhapontigenin, and pinostilbene all had CYP1B1-selective potencies that were similar to resveratrol [134]. More recently, it has been demonstrated that in HaCaT keratinocytes, pterostilbene blocks the nuclear translocation of AhR triggered by specific matter, resulting in the decreased expression of CYP1A1 [135]. Pterostilbene activated Nrf2/HO-1 pathways in asthmatic mice and 16HBE (human bronchial epithelial) cells. No comparison was made with resveratrol [136].

Treatment with piceatannol of NCM460 normal human colon epithelial cells was similarly reported to cause upregulation of phase 2 enzyme expression via activation of the Nrf2 pathway, combined with the inhibition of benzo[a]pyrene-induced CYP1B1 and the stimulation of miR27b-3p expression [137]. Moreover, piceatannol has been shown to act as an agonist for the estrogen receptor alpha in human breast cancer cells [138].

#### 5.2.3. Synthetic Analogs of Resveratrol

Resveratrol has a pleiotropic ability to modulate the carcinogenesis process, but its fast metabolism, low solubility in water, and low bioavailability limit its application. It might also function as a phytoestrogen [139]. Designing synthetic analogs is encouraged by promising data on the natural ones. Methoxylated resveratrol analogs are thought to be more potent AhR pathway modulators and more physiologically stable, with a higher bioavailability and an easier cell uptake [140,141,142].

Accordingly, it was demonstrated that 2,4,3′,5′-tetramethoxystilbene (TMS) was a strong competitive inhibitor of CYP1B1 in an *Escherichia coli* system, with a selectivity that is 50 times greater than that of CYP1A1 [143]. In the human breast cancer cell line, MCF7 TMS effectively inhibited CYP1A1 expression and ethoxyresorufin-O-demethylase, a marker of CYP1A1 activity [144,145]. TMS anticancer activity was confirmed by a 53% reduction in the tumor volume in mice carrying xenografts of MCF7 breast cancer cells [146]. More recent research has further confirmed this activity [147]. In MCF7 cells, TMS more effectively activated AhR and induced the AhR-mediated induction of CYP1A1 activity than did resveratrol and the other tested analogs. TMS reduced the gene expression of IL-8 (3-fold) and increased the gene expression of CYP1A1 (289-fold), CYP1B1 (5-fold), and in MCF7 cells (2-fold), according to gene expression analysis. TMS, in contrast to MCF7 cells, also increased the gene expression of CYP1A1 (5-fold) and CYP1B1 (2-fold) in MCF10A cells but decreased that of NQO1 (1.4-fold). This study also demonstrated that TMS specifically inhibited the cell cycle of tumor MCF7 cells, but not nontumorigenic breast MCF10 cells, in contrast to resveratrol.

In primary human hepatocytes and HepG2 cancer cells, it was demonstrated that DMU-212, a tetra-methoxy analog of resveratrol, was the most potent AhR activator and CYP1A1 inducer among 13 different hydroxy- and methoxy stilbenes, including cis- and trans-resveratrol. Conversely, trans-4-methoxystilbene was not a strong CYP1A inducer in HepG2 cells, but it was one in human hepatocytes. The extensive phase 1 and 2 xenobiotic metabolism in human hepatocytes is likely the cause of differences in stilbene effects between HepG2 cells and human hepatocytes [148]. This suggestion supports earlier studies by Piotrowska-Kempisty et al. [149] demonstrating that CYP1A1 is involved in the metabolic activation of DMU-212 to 3′-hydroxy-3,4,5,4′-tetramethoxystilbene (DMU-214), which exerts more potent cytotoxic effects in ovarian cancer cell and nontumorigenic ovarian cells as compared to the parent compound.

Our research focused on a new class of 3,4-dimethoxy motifs: methoxy-trans-stilbenes. Initial experiments, in which recombinant CYP1A1, CYP1A2, and CYP1B1 were applied, showed that 3,4,2′-trimethoxy-trans-stilbene (3,4,2′-TMS) exhibited an extremely potent inhibitory action against CYP1B1 activity, with an IC50 of 0.004 mM. Moreover, 3,4,2′-TMS demonstrated selectivity for CYP1B1 over CYP1A1 of 90-fold and CYP1B1 over CYP1A2 of 830-fold. Nevertheless, it was found that 3,4,2′,4′-tetramethoxy-trans-stilbene had very little affinity for CYP1A2 and was the most selective inhibitor of CYP1B1 and CYP1A1 [150].

Trans-stilbene compounds with tri-, tetra-, and penta-methoxy groups were studied in nontumorigenic breast epithelial cells and breast cancer cells. The effect of 3,4,2′-trimethoxy (3MS), 3,4,2′,4′-tetramethoxy (4MS), and 3,4,2′,4′,6′-pentamethoxy (5MS) trans-stilbenes on the constitutive expression of CYP1A1 and CYP1B1, as well as the AhR, was evaluated in nontumorigenic MCF10 epithelial breast cell line. The decreased expression of AhR, CYP1A1, and CYP1B1 was observed following the administration of these chemicals. The most significant changes were observed in the case of the AhR. Moreover, 5MS was the most effective inhibitor of the expression of the CYP1A1 and CYP1B1 genes; it decreased the levels of both CYPs′ mRNA transcript and protein from 31 to 89% of their initial levels [151]. In a subsequent study, we evaluated the effect of 3MS, 4MS, and 5MS, compared to resveratrol activity in estrogen-positive MCF7 and estrogen-negative MDA-MB-231 breast cancer cell lines. Remarkably, the expression of AhR in these cells was not significantly altered by any of the stilbenes examined, including resveratrol. However, the CYP1A1 protein level was slightly reduced in MDA-MB-231 cells, while CYP1B1 expression was increased as a result of the treatment with 3MS, but only at the transcript level. On the other hand, 3MS decreased the expression of ERα in MCF7 cells, just like resveratrol did [152]. These findings suggest that resveratrol methoxy derivatives could be effective regulators of the AhR and estrogen metabolism. Furthermore, this impact might be influenced by a number of methoxy groups added to the stilbene structure.

Since the replacement of 4′-hydroxyl with a thiomethyl group is supposed to reduce the toxicity of stilbene derivatives, a series of 4-thiomethyl-trans-stilbene derivatives differing in the number and position of additional methoxy groups were also designed and synthesized and their inhibitory potency against recombinant CYP1A1, CYP1A2, and CYP1B1 was assessed [153]. The most powerful and selective inhibitory effects on CYP1A1 and CYP1B1 activities were shown by the compounds 2-methoxy-4′-thiomethyl-trans-stilbene and 3-methoxy-4′-thiomethyl-trans-stilbene. This suggests that the addition of a thiomethyl group to stilbene derivatives may affect the selectivity and inhibitory potency of the compounds toward P450 isozymes.

The impact of methylthiostilbenes on the Nrf2 signaling pathway was assessed in human keratinocytes (HaCaT cell lines) and mice epidermis [154]. The most potent inducer of GST activity was resveratrol, although all of the methylthiostilbenes tested, namely 3-methoxy-, 3,5-dimethoxy-, and 3,4,5-trimethoxy-4′-methylthio-trans-stilbenes, also increased GST activity. Furthermore, only resveratrol increased the protein level of the GSTP isoform in the mouse epidermis, but the treatment with derivatives and 3,5-dimethoxy-4′-methylthio-trans-stilbene increased the level of GSTM in HaCaT cells. The same effect was observed for GSTP in the case of the compound 3-methoxy-4′-methylthio-trans-stilbene. In HaCaT cells, resveratrol and its derivatives decreased the NQO2 protein level. Therefore, NQO2 inhibition in these cells was possibly associated with elevated GSTP and GSTM expression, as well as an overall GST activity. The findings of this work suggest that resveratrol and its methylthioderivatives activate Nrf2 in human keratinocytes and in mouse epidermis. However, overall, the activity of methylthiostilbenes on the Nrf2 and AhR signaling pathways did not considerably outperform resveratrol′s activity.

Over the last 20 years, the increasing weight of clinical evidence suggests resveratrol can improve human health. Still, more thorough, high-quality clinical trials are needed to move this compound into the clinic. While a clear correlation between the activation and inhibition of these transcription factors has not been discovered, the modulation of the AhR–Nrf2–ER axis is one of its pleiotropic activities. The action of resveratrol depends largely on certain ligands stimulating these pathways and amounts. More effective modulators appear to be synthetic analogs, in particular those with tri-, tetra-, and pentamethoxy groups, especially when it comes to the AhR, Nrf2-, and ER-controlled gene transcription. Further preclinical studies are needed to confirm whether resveratrol has an agonistic or antagonistic effect.

The summary of the modulatory effects of the phytochemicals on the AhR-Nrf2-ER axis described in the text is shown in Table 1.

## 6. Concluding Remarks

The interaction of the AhR with Nrf2 and ER transcription factors affects initiation, promotion, progression, and metastasis, i.e., the major stages of tumorigenesis. The activation of the canonical AhR signaling pathway induces the expression of cytochrome P450 enzymes, which transform inert carcinogens into reactive electrophilic species able to initiate the carcinogenesis process. Moreover, estrogen metabolism catalyzed by CYP1A1 and CYP1B1 leads to the formation of reactive intermediates, such as E2-4-hydroxy derivatives and quinones, which bind to DNA, forming DNA adducts, the important event in tumor initiation. The simultaneous activation of the Nrf2 signaling pathway may protect against DNA and cell damage. Nrf2 signaling is positively modulated by the AhR, but it is inhibited by ERα. Such a crosstalk through altered p300 recruitment to Nrf2-regulated target genes was shown, for e.g., in ER-positive MCF7 breast cancer cells treated with racemic sulforaphane, a dual AhR and ERα activator, 3,3′-diindolylmethane, and TCDD, or E2 [155]. Therefore, the crosstalk between AhR–Nrf2–ERα may protect against carcinogenesis development. The phytochemicals presented in this paper are effective modulators of these pathways, but their effect is usually pleiotropic and depends on the tissues involved. Moreover, as with resveratrol, it often strongly depends on the concentration. Some studies have shown a similar trend for DIM. Synthetic derivatives, such as methoxy stilbenes, seem to be more efficient modulators of these pathways, but the mechanism of their activity in this context requires further studies. Additionally, the fundamental question is how these phytochemicals affect the net AhR–Nrf2–ERα impact in vivo. Such studies are limited and extrapolation of in vitro results may prove clarity because of the extensive metabolism of these phytochemicals in humans.

Studies on aggressive tumors and tumor cell lines have shown that the AhR can be constitutively overexpressed. This leads to the hypothesis that the AhR is chronically activated in tumors, thus facilitating tumor progression. On the other hand, sustained Nrf2 activation in cancer cells is usually observed as a result of an impairment in autophagy, promoting anticancer drug resistance and cancer cell proliferation. Naturally occurring and synthetic stilbenes and *Brassica*-derived phytochemicals, such as I3C and ITCs, may override the Nrf2 survival pathway in cancer cells, resulting in increased apoptosis and cell death.

Dissecting the modulating activity of the AhR–Nrf2–ER axis by phytochemicals, in general, and the classes discussed in this review in preventing cancer initiation/promotion versus the induction of cell killing in advanced cancer cells is still a challenge. Indeed, more selective AhRs and related crosstalk modulators should be searched for.

New approaches, such as the use of cheminformatics, might help to select the most promising compounds within these classes of phytochemicals or their synthetic derivatives.

Given the important role of the AhR and its crosstalk in carcinogenesis targeting these signaling pathways offers the opportunity for improvement cancer prophylaxis and therapy.

## Figures and Tables

**Table 1:** Summary of the modulatory effects of the phytochemicals described in the text on the AhR–Nrf2–ER axis.

Compound	AhR	Nrf2	ER
I3C	- strong agonist in breast cancer cells [75]	- upregulation of Nrf2 expression in breast cancer cells [81]	- downregulation of ERα expression in human tumor cells and breast cancer cells [83,84]
			- downregulation of ERα expression in breast cancer cells [81]
DIM	- weak agonist in rat liver microsomes [76]	- upregulation of Nrf2 expression in breast cancer cells [81]	- downregulation of ERα expression in high doses, upregulation in low doses in endometrial and breast cancer cells [87,88]
			- downregulation of ERα expression in breast cancer cells [81]
ITCs	- inhibition of AhR expression by SFN in MCF7 breast cancer cells [107]	- activation of Nrf2 by PEITC and SFN in vivo (rat liver and kidney) [102] and in vitro (HepG2 cancer cells) [103]	- inhibition of ERα expression by SFN in MCF7 breast cancer cells [106]
	- inhibition of AhR activation by SFN in mice [109]		
Resveratrol	- inhibition of AhR activity (in a concentration-dependent manner) in ER-positive cancer cells [113]	- induction of Nrf2 activation in primary rat hepatocytes [121], K-562 hematopoietic malignant cells [122], hepatic cancer cells [127], and pancreatic cancer cells [128]	- stimulation of ERα expression in breast cancer cells [115]
			- dual effect: estrogen antagonist at high concentrations and estrogen agonist at low concentration in in vivo and in vitro models [116]
Naturally occurring analogs of resveratrol	- inhibition of the nuclear translocation of the AhR by pterostilbene in HaCaT keratinocytes [135]	- activation of Nrf2 by pterostilbene in mice [136] and piceatannol in normal colon epithelial cells [137]	- piceatannol as an agonist of ERα in breast cancer cells [138]
Synthetic analogs of resveratrol	- activation of the AhR by TMS in MCF7 breast cancer cells [147]	- activation of Nrf2 by methylthiostilbenes in human keratinocytes and mouse epidermis [154]	- inhibition of ERα expression by 3MS in MCF7 breast cancer cells [152]
	- activation of the AhR by DMU-212 in primary human hepatocytes and HepG2 cancer cells [148]		
	- inhibition of AhR expression by 3MS, 4MS, and 5MS in breast epithelial cells [151]		

## Data Availability

No new data were created or analyzed in this study.
